# The safety and tolerability of a one strength dose-escalation scheme for subcutaneous immunotherapy with a native house dust mite extract in Chinese children: A multicenter, randomized, open label clinical trial

**DOI:** 10.1016/j.heliyon.2024.e29450

**Published:** 2024-04-10

**Authors:** Lili Zhi, Yan Bai, Wang Liao, Guohua Chen, Tingting Gao, Xia Wan, Jiawen Liang, Lingling Liu, Liang Chen, Wenna Zhang, Jun Bai

**Affiliations:** a Department of Allergy, The First Affiliated Hospital of Shandong First Medical University, Jinan, 250014, China; b Department of Pediatrics, Union Hospital, Tongji Medical College of Huazhong University of Science & Technology, Wuhan, 430022, China; c Department of Pediatrics, Affiliated Foshan Maternity & Child Healthcare Hospital, Southern Medical University, Foshan, 528000, China

**Keywords:** Subcutaneous immunotherapy, Children, Safety, Accelerated dose escalation, House dust mites

## Abstract

**Background:**

Allergen immunotherapy (AIT) is still the only treatment that may affect the natural cause of allergic disease. This study is to investigate whether an accelerated up-dosing scheme for subcutaneous allergen immunotherapy (SCIT) using a native house dust mite (HDM) allergen extract is as safe as the standard 3-strengths dose-escalation scheme in children with moderate to severe allergic rhinitis or rhinoconjunctivitis with or without asthma in China.

**Methods:**

In this multicenter, open label, randomized controlled trial, the children aged 5–14 years were randomized 1:1 either to One Strength group or the Standard group. The dose escalation scheme for patients in the One Strength group included 6 injections of strength 3, whereas the Standard group comprised 14 injections using strength 1, 2, and 3. All treatment-emergent adverse events (TEAEs) were recorded and analyzed. The 5-point Likert scale was used to assess tolerability (ChiCTR2100050311).

**Results:**

Overall, 101 children were included in the Safety Set (One Strength group: 50 vs. Standard group: 51). A total of 26 TEAEs were reported for 15 children. TEAEs related to AIT occurred in 10 % of the children in the One Strength group and 11.8 % of the Standard group. The number of systemic adverse reactions was comparable in both groups (One Strength: 5 vs. Standard: 4). No serious TEAEs was recorded for either group. 90.0 % of patients in the One Strength group reached the maintenance dose without an interventional dose adjustment due to adverse events, compared to 78.4 % in the Standard group. All patients who completed the dose-escalation phase reached the recommended maintenance dose of 1.0 ml of strength 3.

Investigators and patients rated the tolerability of the One Strength regimen slightly better than the Standard scheme.

**Conclusions:**

This exploratory study suggests that the accelerated One Strength dose-escalation scheme is comparable in safety and tolerability to the Standard regimen. However, due to the preliminary nature and small sample size, further research with larger sample sizes and robust study designs is necessary for confirmation.

## Key message

There have been rare safety data on accelerated dose-escalation schemes in Chinese children especially when using only the highest strength of native SCIT products. Therefore, the current multicenter, randomized, open label clinical trial investigated the safety and tolerability of a One Strength dose-escalation scheme with a native HDM SCIT product in children suffering from HDM-allergic rhinitis or rhinoconjunctivitis with or without asthma in China.

## 1 Introduction

Allergic rhinitis (AR) and asthma are the most common airway allergic diseases, characterized by an inflammatory response mediated by immunoglobulin E (IgE) in allergen-sensitized individuals [1]. The prevalence of AR was up to 40 % among children worldwide [2]. In China, the reported prevalence of AR in children ranges from 9.8 to 22.4 % [3]. Asthma is a chronic and heterogeneous respiratory disease with an estimated prevalence of 4.3 % worldwide [4]. One recent study has shown a prevalence of asthma in Chinese children of about 3 %. However, more than 50 % of asthmatic children have co-morbidities of AR [5], leading to a considerable psychological and economic burden [[6], [7], [8]]. The house dust mites (HDM) *Dermatophagoides farinae (Der. f.)* and *Dermatophagoides pteronyssinus (Der. p.)* are the most common aeroallergen triggers associated with persistent perennial symptoms of patients with AR and allergic asthma (AA) in China [3]. A cross-sectional survey of Chinese patients with asthma and rhinitis showed that patients were highly sensitized to *Der. f.* (59.0 %) and *Der. p.* (57.6 %) [9], while another study indicated 83.7 % of patients were concomitantly sensitized to *Der. f.* and *Der. p.* [10].

AIT has been used to treat allergic diseases caused by inhalant allergens for many decades and it is an effective treatment for patients with seasonal or perennial allergic rhinoconjunctivitis and/or asthma. Up to now, AIT is still the only treatment that may affect the natural cause of allergic diseases, and it can also prevent the development of asthma in patients with allergic rhinitis [11]. In China, AIT has been in use for only 20 years, and so far, there are only natural mite extracts available in clinical practice. Novo Helisen® Depot (NHD) house dust mite products have received regulatory approval in China in 1999 as the first HDM product for subcutaneous AIT (SCIT). The standard dose-escalation scheme or similar dosing schemes using NHD strengths 1 to 3 are safe and efficacious in the treatment of HDM allergy [[12], [13], [14]]. However, there is a need of improvement concerning the patients' adherence which is known to affect the efficacy of AIT [15]. It has been reported that the inconvenience required for the injection being a major cause of the nonadherence, while systemic reactions can also lead to discontinuation of SCIT [16,17]. Therefore, optimal dose-escalation regimens will increase the convenience for the patients by minimizing the number of injections and hence the number of visits to the physician's practice. Meanwhile, the determination on the adverse event per injection which may lead to a subsequent dose adjustment should also be considered in optimal dose-escalation regimens. Cluster and rush immunotherapy have also been applied to accelerate the dose-escalations of AIT, but there are still concerns on the unacceptably high rate of SRs associated these two methods [18]. Moreover, the number of injections of cluster and rush immunotherapy has not been reduced and the multi-injections in one day will cause the difficulty in the determination on the occurrence of adverse even per injection thereby hindering the dose adjustment. In recent years, safety and tolerability of accelerated dose-escalation schemes for several SCIT preparations were performed especially for adolescents and adults in prospective comparative trials [[19], [20], [21], [22], [23], [24]]. However, there have been rare safety data on accelerated dose-escalation schemes in children especially when using only the highest strength of native SCIT products. Therefore, the present trial investigated the safety and tolerability of a One Strength dose-escalation scheme with a native HDM SCIT product in children suffering from HDM-allergic rhinitis or rhinoconjunctivitis with or without asthma in China.

## 2 Methods

### 2.1 Study design

This prospective, multicenter, open label, randomized and controlled trial was conducted between September 2021 and February 2023 in 3 hospitals in China.

The main inclusion criteria were: male or female outpatient children, 5–14 years of age; HDM-induced IgE-mediated moderate to severe allergic rhinitis or rhinoconjunctivitis (classified according to Allergic Rhinitis and its Impact on Asthma (ARIA) [25]), with or without asthma, classified as “well controlled” according to Global Initiative for Asthma (GINA) guideline [26]; confirmed diagnosis of HDM allergy based on a positive skin prick test result (wheal for house dust mites (*Der. f.* and/or *Der. p.*) ≥ 3 mm in diameter, histamine wheal ≥3 mm, sodium chloride (NaCl) control reaction <2 mm) and HDM-specific IgE ≥0.70 kU/L; symptoms of allergic rhinitis or rhinoconjunctivitis triggered by HDM exposure over at least the last 1 year; treatment with symptomatic anti-allergic medication for at least 1 year prior to enrollment.

Exclusion criteria included a history of confirmed anaphylaxis after an SCIT injection within the last 5 years, current treatment with any kind of AIT, and uncontrolled/partly controlled asthma according to the GINA guideline [26]. Moreover, patients with autoimmune diseases, β-blocker use, and contraindication for the use of epinephrine could not be enrolled.

This study has been approved by the ethical committees of all participating hospitals. Written informed consent to participate in the study was obtained from each patient's parent or guardian before any study-related activities were performed. This study was registered at www.chictr.org under the identifier ChiCTR2100050311 (A multicenter, randomized, open clinical study to evaluate the safety of a single concentration accelerated dose escalation regimen using semi-depot house dust mite allergen extract in the treatment of moderate-to-severe allergic rhinoconjunctivitis in Chinese children with or without asthma) (Aug-26-2021).

### 2.2 Treatment

**Fig. 1:**
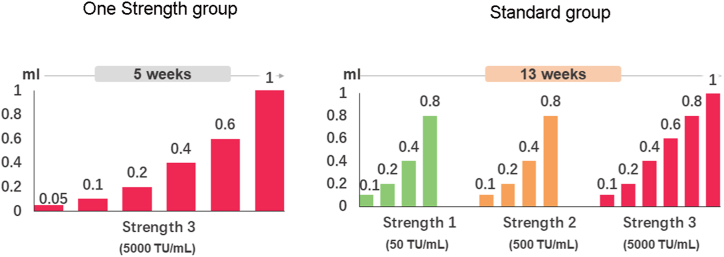
Dose-escalation schemes during SCIT with the native house dust mite product in the One Strength or Standard group.

Novo-Helisen® Depot [NHD (*Dermatophagoides pteronyssinus*: *Dermatophagoides farinae* 50:50 % by volume) (manufactured by Allergopharma GmbH & Co. KG, Reinbek, Germany) is an aluminum hydroxide-adsorbed house dust mite SCIT product. Patients were randomized 1:1 into the One Strength group receiving 6 injections with increasing doses of only strength 3 (5000 TU/mL) (Fig. 1), followed by 2 injections with the individual maximum tolerated dose, or the Standard group receiving 14 injections with increasing doses of three different strengths (strength 1: 50 TU/mL; strength 2: 500 TU/mL; strength 3: 5000 TU/mL) (Fig. 1), followed by 2 injections with the maximum tolerated dose. SCIT injections were administered subcutaneously into the patient's extensor side of the upper arm, a hand's breadth above the elbow. All patient was to be kept under close supervision by a qualified and trained investigator for at least 180 min after each injection. Dose adjustment was performed if local or systemic adverse reactions occurred based on a predefined regime. The duration of the treatment was approximately 16 weeks for patients randomized to One Strength group and 24 weeks for patients of the Standard group.

### 2.3 Assessment of safety and tolerability

Safety and tolerability end points focused on treatment-emergent adverse events (TEAEs), defined as any adverse events (AE) that started or worsened after the first injection of trial medication until 30 days after the last administration of trial medication or trial-related procedure. An adverse drug reaction was defined as all untoward and unintended responses to the AIT related to any dose administered. Local adverse reaction was defined as AE related to the AIT and occurring at the injection site. Intense local adverse reaction were defined as reactions at the injection site that led to swelling of >10 cm or caused distinct discomfort that interfered with the patient's daily activities, or after systemic reactions, which were graded based on organ systems involved and severity according to the World Allergy Organization (WAO) Subcutaneous Immunotherapy Systemic Reaction Grading System [27].

Systemic adverse reaction was defined as AE related to the AIT and graded according to the World Allergy Organization (WAO) grading system based on the organ systems involved and the severity of the reaction [27]. The assessment of the intensity (severity) of an AE was based on the following criteria: Mild: Transient symptoms, no interference with the patient's daily activities. Moderate: Marked symptoms, moderate interference with the patient's daily activities. Severe: Considerable interference with the patient's daily activities. Overall tolerability was assessed by the investigators and the patients using a 5-point Likert scale (1 = very bad; 5 = very good) [28]. Moreover, changes in laboratory values (hematology, clinical chemistry, and urinalysis) measured before and after the treatment phase, changes in vital signs and lung function measured before and after the treatment phase were documented.

### 2.4 Statistical analysis

The study was designed as an exploratory study with no formal estimation of sample size. Initially, 35 patients per each group would guarantee a probability of 95 % that AEs with a true incidence rate of 8.6 % occur at least once in each group [29]. Considering drop-out rate, 50 patients in each group, 100 patients in total will be enrolled. The main analysis of safety is performed for the safety set (SAF): patients who received at least 1 dose of NHD. Patients experiencing major protocol deviations were not excluded from the SAF. Missing values were not imputed, except missing start time of TEAEs. Continuous data were summarized by descriptive statistics (number of observations, number of missing observations, mean with 95 % confidence interval [CI] according to the t-distribution, standard deviation [SD], minimum, maximum, 1st and 3rd quartile and median). Categorical variables were summarized by absolute and relative frequencies. The groups were compared with adequate exploratory statistical tests. All CIs and statistical tests were calculated in an exploratory way using a two-sided significance level of α = 0.05.

## 3 Results

### 3.1 Basic demographic and characteristics

**Fig. 2:**
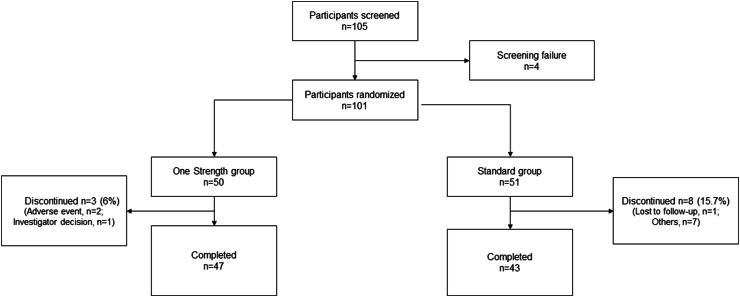
Patient flowchart. Note: Investigator's decision: The patient had been using traditional Chinese medicine for immune regulation, yet the investigator was unfamiliar with the ingredients of this treatment. Prioritizing safety, the patient was granted permission to withdraw from the study. Others: six patients were withdrawn from the study due to COVID-19 control measures, while another patient was withdrawn due to non-compliance.

Overall, 105 children were enrolled, of which 4 patients (3.8 %) were screening failures. A total of 101 patients were randomized, 50 patients to the One Strength group and 51 patients to the Standard group. All of them received at least 1 SCIT injection and were included in the SAF. Finally, 11 patients (10.9 %) terminated the trial prematurely, 3 of them from the One Strength group (2 due to non-serious AIT-related TEAEs, 1 due to the investigator's decision) and 8 from the Standard group (2 due to loss during follow up or because of poor compliance and 6 due to COVID-19 pandemic restrictions) (Fig. 2).

The demographic and baseline characteristics were comparable between the two groups apart from the age distribution since the children in the One Strength group were significantly older than in the Standard group (7.9 ± 2.44 vs. 6.7 ± 2.16, respectively, p = 0.0034). Table 1 shows baseline demographic and clinical characteristics of the SAF.

**Table 1:** Baseline demographic and clinical characteristics of patients in the Safety Set.

Variable	One Strength group (n = 50)	Standard group (n = 51)	*P* value
Age, years			0.0034^a^
Mean (SD)	7.9 (2.44)	6.7 (2.16)	
Median (range)	8.0 (5–14)	6.0 (5–13)	
Sex, n (%)			0.4389^b^
Male	35 (70.0)	32 (62.7)	
Female	15 (30.0)	19 (37.3)	
BMI (kg*/*m^b^), mean (SD)	15.79 (2.426)	16.00 (3.116)	0.9783^a^
Asthma diagnosis, n (%)			0.0860^b^
With asthma	24 (48.0)	33 (64.7)	
Without asthma	26 (52.0)	18 (35.3)	
Percentage of tested FEV1 of predicted value (%), mean (SD)	96.90 (12.393)	94.70 (14.673)	0.2817^a^
Total IgE, median (range)	410.650 (47.2–2500)	407.850 (16.26–2500)	0.5860^a^
Specific IgE for *Der p.*, median (range)	34.400 (0.45–135)	39.900 (0.82–120.53)	0.9429^a^
Specific IgE for *Der f.*, median (range)	55.050 (0.78–174.94)	60.400 (0–159.79)	0.9047^a^

SD, standard deviation; BMI, body mass index; FEV1, Forced Expiratory Volume in the first second; *Der p., Dermatophagoides pteronyssinus*; *Der f., Dermatophagoides farinae*.

a p-value from the two-sided van Elteren-Test comparing both treatment groups.

b p-value from the two-sided Mann Whitney *U* test comparing both treatment groups.

Median treatment duration was 78 days in the One Strength group and 141 days in the Standard group. From the SAF, 45 of 50 patients (90.0 %) in the One Strength group and 40 of 51 patients (78.4 %) in the Standard group reached the maintenance phase without dose adjustment. Overall, 16 (15.8 %) patients did not reach the maintenance phase due to TEAEs (One Strength: 2; Standard: 6) or premature discontinuation (One Strength: 3; Standard: 5). All patients who completed the dose-escalation phase reached the maintenance dose of 1.0 ml of strength 3.

### 3.2 Safety

Overall, 15 patients (14.9 %) experienced at least one TEAE. The proportion of patients with at least one TEAE was comparable between the two groups (One Strength: 7 patients (14.0 %); Standard: 8 (15.7 %); p = 1.0000). 26 TEAEs occurred in total, 8 in the One Strength group and 18 in the Standard group. No death or no serious AEs were reported (Table 2). Investigators assessed 15 of the 26 TEAEs to be related to AIT which occurred in 11 patients (10.9 %). The percentage of patients with at least one AIT-related TEAE was comparable in both groups, while the number of events was slightly higher in the Standard group than in the One Strength group (One Strength: 10.0 % of patients, 6 events vs. Standard: 11.8 % of patients, 9 events; p = 1.000, for the number of patients affected). In addition, the ratio of AIT-related TEAEs per patient was comparable between two groups (One Strength: 1.2 vs. Standard: 1.5). No events related to trial procedures was observed. A total of 93.3 % of AIT-related TEAEs were reported to be of mild intensity, 6.7 % of AIT-related TEAEs was of moderate severity.

**Table 2:** Number and proportion of patients in the Safety Set experiencing treatment emergent adverse events (TEAEs), local and systemic adverse drug reactions and number of respective events observed in the One Strength and Standard group.

	One Strength group (n = 50)		Standard group (n = 51)		*P* value
	Patients, n (%)	Events, n	Patients, n (%)	Events, n	
TEAEs	7 (14.0)	8	8 (15.7)	18	1.0000^a^ 1.0000^b^
TEAEs related to AIT	5 (10.0)	6	6 (11.8)	9	1.0000^a^ 1.0000^b^
Mild severity	5 (10.0)	5	6 (11.8)	9	1.0000^a^ 1.0000^b^
Moderate severity	1 (2.0)	1	0 (0)	0	0.4950^a^ 0.4950^b^
Severe severity	0 (0)	0	0 (0)	0	NA^a^ NA^b^
Local adverse reactions	1 (2.0)	1	4 (7.8)	5	0.3624^a^ 0.3624^b^
Systemic adverse reactions	5 (10.0)	5	2 (3.9)	4	0.2691^a^ 0.2691^b^
WAO grade 1	4 (8.0)	4	0 (0)	0	0.0564^a^ 0.0564^b^
WAO grade 2	1 (2.0)	1	2 (3.9)	4	1.0000^a^ 1.0000^b^
AIT-related TEAEs leading to discontinuation	2 (4.0)	2	0 (0)	0	0.2426^a^ 0.2426^b^
Systemic adverse reactions leading to dose reduction	2 (4.0)	2	2 (3.9)	4	1.0000^a^ 1.0000^b^
Serious TEAEs	0 (0)	0	0 (0)	0	NA^a^ NA^b^

NA; not applicable; TEAE, treatment-emergent adverse event.

a p-value from the two-sided van Elteren-Test comparing both treatment groups.

b p-value from the two-sided Mann Whitney *U* test comparing both treatment groups.

Six reported TEAEs related to AIT were local adverse reactions (One Strength: 1 event in 1 (2.0 %) patient vs. Standard: 5 events in 4 (7.8 %) patients) with no significant difference between the two groups (p = 0.3624, for the number of patients affected).

**Fig. 3:**
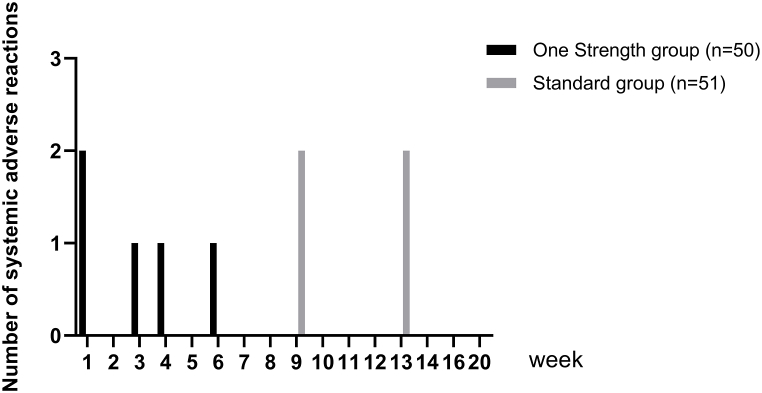
Number of systemic adverse reactions per week during SCIT with the native house dust mite SCIT product using the One Strength or Standard dose-escalation scheme.

In total, 9 systemic adverse reactions were observed in 7 (6.9 %) patients. Five systemic adverse reactions were reported for 5 patients of the One Strength and 4 for 2 patients of the Standard group (p = 0.2691) (Table 3). The ratio of systemic adverse reaction per patient was lower in the One Strength than in the Standard group (One Strength: 1.0 vs. Standard: 2.0). Four of the systemic reactions were classified as WAO grade 1 reactions which occurred in 4 patients of the One Strength group while 4 systemic reactions occurring in 2 patients of the Standard group and 1 systemic reaction in 1 patient of the One Strength group were classified as WAO grade 2. No events of WAO grade 3 or higher were reported. All nine systemic reactions were assessed as non-serious, and all occurred with strength 3 during the dose-escalation phase (Fig. 3).

**Table 3:** Type of adverse reactions related to AIT. System Organ Class and Preferred Terms.

	One Strength group (n = 50)		Standard group (n = 51)		P value
	Patients, n (%)	Events, n	Patients, n (%)	Events, n	
General disorders and administration site conditions	1(2.0)	1	4(7.8)	5	0.3624^a^ 0.3624^b^
Injection site swelling	1(2.0)	1	0(0)	0	
Injection site pruritus	0(0)	0	1(2.0)	2	
Injection site rash	0(0)	0	3(5.9)	3	
Respiratory, thoracic and mediastinal disorders	5(10.0)	5	2(3.9)	2	0.2691^a^ 0.2691^b^
Asthma exacerbation	1(2.0)	1	0(0)	0	
Cough	4(8.0)	4	2(3.9)	2	
Skin and subcutaneous tissue disorders	0(0)	0	2(3.9)	2	0.4950^a^ 0.4950^b^
Pruritus	0(0)	0	1(2.0)	1	
Rash	0(0)	0	1(2.0)	1	

a p-value from the two-sided van Elteren-Test comparing both treatment groups.

b p-value from the two-sided Mann Whitney *U* test comparing both treatment groups.

### 3.3 Time to onset of AIT-related TEAEs

**Fig. 4:**
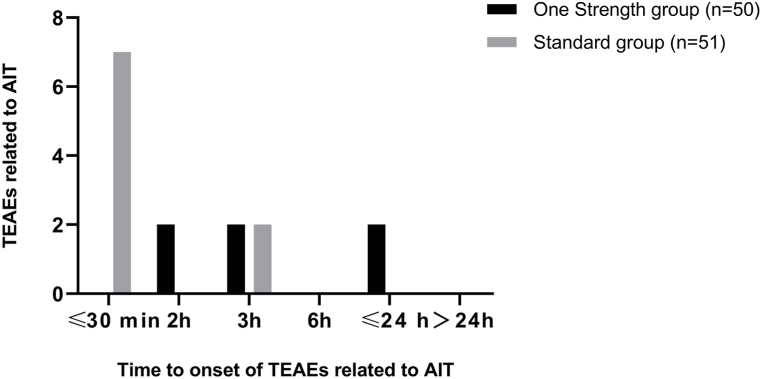
Time to onset of TEAEs related to AIT during SCIT with the native house dust mite SCIT product using the One Strength or Standard dose-escalation scheme.

In the One Strength group, 4 of the 6 AIT-related TEAEs occurred within 3 h after AIT and 2 AIT-related TEAEs within 24 h. All AIT-related TEAEs (9 of 9 events) reported for the Standard group occurred within 3 h after SCIT administration (Fig. 4).

### 3.4 Tolerability and other safety parameters

**Fig. 5:**
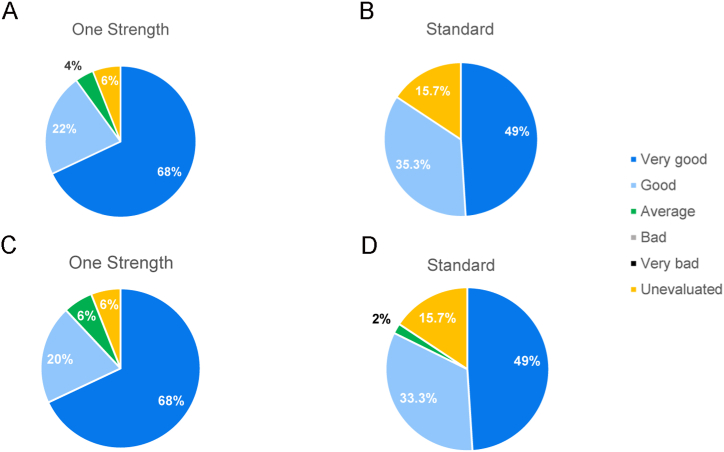
Assessment of overall tolerability of SCIT with the native house dust mite product in the One Strength and Standard group by the investigators (**A, B**) and the patients (**C, D**) after the escalation phase on a 5-point Likert scale (very bad - bad - average - good - very good). (One Strength group, n = 47; Standard group, n = 43).

Investigators assessed tolerability as good or very good for 90 % of patients in the One Strength and for 84.3 % of patients in the Standard group (Fig. 5A and B), whereas 88 % of patients in the One Strength and 82.3 % of patients in the Standard group assessed tolerability for themselves as good or very good (Fig. 5C and D).

No clinically significant changes from baseline for any observed laboratory parameter were reported. The clinical chemistry, hematology, and urinalysis values showed no relevant changes in any parameter for any group. Vital signs and lung function at the final visit did not show clinically significant changes from baseline.

## 4 Discussion

In current study, it was demonstrated that the dose-escalation period of SCIT with the native HDM product can be accelerated in a One Strength regimen using only strength 3 with 6 injections in Chinese children. The safety profile was acceptable and did not reveal unexpected findings, the tolerability was good and both parameters were overall comparable to those of the standard dose-escalation scheme.

The incidence of patients experiencing TEAEs was comparable in both groups which was in accordance with previous studies [19,24]. It is reported that local adverse reactions from AIT are common during the up-dosing and maintenance phase [30].

In this study, both, the number of reported events and the number of patients affected by local reactions, were less for the One Strength group than the Standard group, which might be caused due to the less injections in the One Strength group. The number of local adverse reaction was generally lower compared to data from previous studies, showing that as many as 26–86 % of the patients receiving SCIT experience local adverse reactions [31]. All the local adverse reactions did not lead to the discontinuation or dose reduction, which was similar with results of trial in adults [24].

Systemic adverse reactions occurred in about 6.9 % of all the patients and were reported in more patients of the One Strength group (10.0 % vs. 3.9 %). These results are in line with data reported in adults and children [19,23,24,31]. However, the intensity of the systemic adverse reactions seemed lower in One Strength group. There were 4 events of WAO grade 1 and 1 event of WAO grade 2 in One Strength group, while all the 4 systemic adverse reactions in Standard group were classified as WAO grade 2. All reported TEAEs were of mild or moderate intensity, and no TEAE was classified as severe. The frequency and the intensity of TEAEs were overall not considered a safety concern, and no change in the benefit risk profile of the medicinal product was regarded necessary, which was consistent with other trials [19,24,32]. All the systemic adverse reactions occurred in the escalation phases with the Strength 3, therefore, the one strength escalation scheme skipping Strength 1 and 2 was feasible.

Overall, 90.0 % (45/50) of patients in the One Strength group reached the maintenance dose without dose adjustment due to adverse events, compared to 78.4 % (40/51) in the Standard group. Similar with previous study, all AIT-related TEAEs leading to dose reductions occurred during the dose-escalation period [33], which also suggested that all the patients completed the dose-escalation phase can achieve the target dose concentration.

Though it has been reported that AIT using the standard dose-escalation scheme is an efficacious and safe treatment option in children [34], the use of alternative dose-escalation schemes with fewer injections is increasingly common in daily practice. For all patients in our trial, the median treatment duration was reduced in the One Strength group, meanwhile the numbers of injections and the Strength used were also reduced. Therefore, the advantage of a shortened AIT therapy is that administration of fewer injections is comfortable and more convenient for the patients having fewer visits in doctor's practice. In addition, less absenteeism from school or from leisure activities of children and adolescents is especially relevant for pediatric patients. The standard dose-escalation scheme takes 13–14 weeks, while the One Strength dose-escalation scheme needs only 5 weeks, which can save about 2 months to achieve the maintenance dose. Fourteen to fifteen injections are needed for the standard dose-escalation scheme, while only 6 injections are needed for One Strength dose-escalation scheme. For the Strength used, the standard dose-escalation scheme will need 3 to 4 Strength, while the One Strength dose-escalation scheme only needs Strength 3, which can reduce the risk of confusion at different strengths, facilitate the management of AIT, and facilitate the interpretation of adverse reactions for dose adjustment. Though the cluster and rush build-up for aeroallergen immunotherapy can also reduce the treatment duration [18], they cannot reduce the numbers of injections and the strength needed. The present results confirm the previously generated data that has already led to an approval of the shorter dose-escalation scheme in adults in western countries [21].

The overall tolerability at the end of the escalation phase was assessed as “very good” or “good” by the majority of investigators and patients, which were in accordance with previous trials in adults and children [19,24,35].

There are several strengths of this study. In Europe, primarily allergoid preparations are used for SCIT, while in China only native allergen preparations are available. This is the first randomized controlled investigating safety of a One Strength dose escalation scheme in children suffering from HDM-allergic rhinitis or rhinoconjunctivitis with or without well controlled asthma in China. Moreover, this is the first trial investigating One-strength dose-escalation in HDM allergic children. Most trials investigating accelerated dose-escalation focus on adults or just include adolescents [[20], [21], [22],^24^], while only a grass pollen allergoid was shown to be safe and tolerable in children too when used in a One Strength regimen [19]. Up to now, no accelerated dose-escalation regimen of any native HDM SCIT preparation was shown to be safe and tolerable in China. Further studies should aim to show safety of the One Strength dose-escalation scheme in adolescents and adults as well as patients only suffering from HDM-allergic asthma.

In conclusion, the One Strength dose-escalation scheme with 6 injections of an unmodified HDM SCIT preparation is as safe and tolerable as the Standard dose-escalation scheme in Chinese children with moderate to severe allergic rhinitis or rhinoconjunctivitis with or without asthma. The accelerated dose-escalation scheme offers the opportunity for a faster up-dosing period which could help to increase adherence to SCIT.

## Funding information

This study received a grant from Beijing Hengji Health Management and Development Foundation. The grant was used for data management and statistics (This work was done by Beijing E-Shine Biopharmaceutical Technology Co.Ltd). Grant number: HJKY2021001-001.

## Data availability statement

Data will be made available on request.

## CRediT authorship contribution statement

**Lili Zhi:** Writing – review & editing, Writing – original draft, Methodology, Investigation, Formal analysis, Data curation. **Yan Bai:** Validation, Resources, Investigation. **Wang Liao:** Software, Resources, Investigation, Formal analysis, Data curation. **Guohua Chen:** Software, Resources, Investigation. **Tingting Gao:** Validation, Investigation, Data curation. **Xia Wan:** Investigation, Formal analysis, Data curation. **Jiawen Liang:** Resources, Investigation. **Lingling Liu:** Investigation. **Liang Chen:** Software, Investigation. **Wenna Zhang:** Resources, Investigation. **Jun Bai:** Supervision, Resources, Investigation, Funding acquisition, Conceptualization.

## Declaration of competing interest

The authors declare that they have no known competing financial interests or personal relationships that could have appeared to influence the work reported in this paper.
